# Transactions of the Medical Association of Georgia, 1884

**Published:** 1885-01

**Authors:** 


TRANSACTIONS OF THE MEDICAL ASSOCIATION OF GEORGIA.—Thirty-fifth Annual Session, 1884. James P. Harrison & Co., Atlanta, Ga.: Publishers.

I regard this as perhaps, the most valuable issue of transactions in the history of the Association. Dr. James A. Gray, the Secretary, and the publisher, both deserve the thanks of the profession for the parts they have each so well maintained in the work; the

*Wood recommends Hoffman's anodyne and spts. æth. nitrosi for restlessness; and musk, asafœtida, camphor and similar drugs for the hiccough."

first for the general style of the book and judicious selection of matter for publication, and the second for its handsome typography.

Several of the papers contributed are of unusual and remarkable value. They are worthy of a much wider circulation than they will get in the Transactions. This prompts me to give some of them such a review for THE JOURNAL, as I have time for such work.

First, I am pleased to notice the very happy welcome delivered by Dr. Wm. F. Holt, of Macon, and the no less happy response by Dr. L B. Alexander, of Forsyth. These gentlemen were obviously in the performance of congenial work.

The annual address by Dr. A. W. Calhoun, the President, should be read and closely studied, not by the physicians of Georgia only, but by every parent, teacher, guardian of youth, and educational board, as well. The subject is School Hygiene, in Relation to its Influence upon the Vision of Children; or, School Sanitation. "Education," says the Doctor in this admirable address, "is the preparation for the work of life, not a thing that is good in itself. If it has helped life to be healthy, happy, successful and long, then it has been good; if, in any degree, it has caused disease, unhappiness, non-success, then it has been bad."

It is seen from the title that the speaker confined his remarks chiefly to the influence of school life and regimen upon the vision of children. But this is only a single topic from the many equally urgent that could be discussed under the prolific head of School Sanitation. Here is a field for study and investigation that is beginning to attract the serious attention of every humane physician, and rightly informed citizen in this country. Never in the world's history has popular education been so advanced and pushed as now.

But all popular enthusiasm tends constantly towards dangerous extremes; and is not this too true already in the United States? The people are crazy on education, forgetting, as President Calhoun so justly remarked, it is only a good as it achieves good results. In defiance of this, it would seem, the most hurtful methods are all the vogue.

Dr. Calhoun has done his part for the eye in behalf of the children, now let others follow; one, school treatment of spine; another on the brain and nervous system of over worked children at school, and yet another on school-room development of the chest and lungs.

On the 3d day of the session, Dr. H. V. M. Miller, of Atlanta, read an able paper, The Effects of Attitude in the Treatment of Consumption. This was heard with fixed attention, but unfortunately does not appear in the Transactions. I desire here, however, to notice some remarks made in the discussion of the paper, by Dr. Bizzell, of Atlanta, who lifted the subject at once out of the murky atmosphere of mere Kochism.

Dr. Bizzell said:

"Consumption is a disease whose etiology lies deeper than mere tubercular deposit or destruction of lung structure.

"It may be, and in all likelihood is true, that there is a germ or micrococcus that is capable of setting up that peculiar pathological process which we call consumption or tuberculosis. Just as there is a seed for the familiar plant we call cotton, but all observation and experience teach us that in order to get the plant to bear fruit and flourish, the condition of soil, climate, rainfall, etc., must be suited to the plant, or failure will result. Just so with certain of the infectious diseases, radically change the environment and the disease is jugated.

"If tuberculosis be an infectious disease resulting from the implantation of disease germs, as the experiments of Dr Koch seem to demonstrate, a careful study of the disease will show that, like many of the seeds of plants and spores of fungi in the vegetable world, it requires a certain preparation of soil, as it were, and certain forming conditions, or otherwise the disease seems to lie dormant, loses its effect and the victim escapes; else how are we otherwise to account for the very general immunity of nurses and friends or companions of the sufferers, notably those in attendance constantly on this class of patients as the nurses of the Brompton Hospital for consumptives in England, etc.

"It is a matter of common observation that phthisis always declares itself after the organism has attained a condition of low vitality, whether it be by inheritance or a feeble organism struggling into puberty, or an originally fair constitution hampered by unsanitary environment, abuse of the vital powers, overwork, anxiety of mind, indigestion. No matter what route the individual may have traveled or what his original endowment of constitutional vigor, before phthisis declared itself, he must have reached a low plane of vitality before the disease can fully and clearly establish itself. The foregoing being in the main propositions which, on investigation, resolve themselves into facts, we must reach the conclusion that there can be no rational treatment for the cure or prevention of consumption that is not hygienic. To improve the powers of digestion and assimilation, and in consequence produce rich blood and healthy cell growth, if we cannot attain to this we cannot hope for arrest in the progress of the disease, much less for cure. It matters not how we reach this much to be desired result, it is sufficient if we attain it. It cannot be effected by the administration of drugs alone, even though they partake of the nature of food as cod liver oil, for my experience, no doubt, accords with that of the majority present when I say that in not more than one case in a hundred will cod liver oil be accepted and digested by the stomach without previous preparation of that organ through hygienic methods. And of all hygienic methods there is nothing that does not include fresh air, pure water and muscular exercise; temperature and humidity, also, are of very great importance, and the barometric pressure is not to be lost sight of. As has been pointed out by Dr. Miller, the mountain region of the Blue Ridge and Alleghanies, which lie almost at our doors, will supply all that is necessary in the hygienic treatment of phthisis. There is no disease to which we should apply more literally the precept of the Great Teacher, "What thou doest, do quickly," nor to which we can apply with greater force the Latin motto, "*obstu principes*," oppose the beginnings. For if the golden opportunity has been allowed to go by and the destruction of lung structure has already advanced to the formation of cavities, further treatment as a rule is only palliative."

There is more good sense and sound philosophy in these words of the Doctor than in all the micromaniac dreams of Koch and his school.

The paper contributed by Dr. Eugene Foster, of Augusta, "Syphilis as a Sociological Problem," in which he triumphantly exposes the false opinions and statements of Herbert Spencer, is one of the ablest contributions to science and humanity that has been published in many a day. When the philosopher of grand social problems stepped into the domain of medicine, he was obviously out of his sphere, and Dr. Foster takes his photograph in the midst of his scientific diversions, which makes it painfully obvious.

The article is replete with valuable information, even to the profession, as regards the most loathsome and interesting malady of modern times.

I especially commend it to the large class of admirers of Spencer, who hang upon his notoriously unscriptural views of social science as a latter-day gospel.

Dr. R. J. Nunn, of Savannah, has a very valuable article on "Diet in Consumption and other Morbid States." I quote the following as the key-note of the part devoted to consumption; it, too, has the right ring, and is not hazy with micrococci: "Therefore for the development of tuberculosis, two conditions are necessary:

"1st. A definite soil.

"2d. An indefinite irritant.

"The reaction of the soil is always the same under the influence of any irritant, whether that irritant be a baccillus or not, since the result (tuberculosis) following a lesion in such a soil depends upon the character of the soil, and not upon the character of the irritant, even though one irritant, say bacilli, may act more readily than other irritants. In view of the demonstrated fact that simple injuries of any kind can excite a tuberculosis, but only in certain individuals and tissues, it is evident that tuberculization is determined by the kind of soil, and not by a specific irritant. Tubercle should, therefore, be defined as being an inflammatory new formation in a special individual or tissue.

"What is the place for tubercle in pathology? The anatomical criterion for tubercle is a granulation tissue made up of lymphoid or epithelioid cells, which, on account of deficiencies in the soil. does not undergo any higher organization, nor tend to heal, but tends to form nodes and undergo cheesy changes. Under favorable circumstances it may heal through fibroid change (*H. F. Formad, Jour. Am. Medical Association, No.* 6, *p.* 144, *et seq.*)"

I regret that so little time and space are left me to speak of Dr. Gaston's able article on "The Pathology and Therapeutics of Diseases of the Nerve Centres." I can only give the condensed teaching: "The fountain head of energy for all the functions lies in the nerve centres; and by controlling emanations from this source of power the vital forces will be propagated with regularity and uniformity to all the remote parts of the physical organization, and it is to this we must look for the cure of diseases.

"The means, then, to be adopted for averting injurious impressions upon the nerve centers, and the measures to be used for the correction of their derangements, make up the whole prophylactic combination of hygiene, and include all therapeutic agency in the treatment of diseases, let the details be varied as they may. Rigid clinical observation should reveal the source of the disturbances in the physical organization, and the proper means of correcting them by regimen and medication."

There are other valuable contributions which, for want of space, I cannot refer to. J. H. L.

